# Supplementary figures and images for: A new clinical prognostic nomogram for liver cancer based on immune score
Source: PLoS One. 2020 Jul 30;15(7):e0236622. doi: 10.1371/journal.pone.0236622 (PMC7392298; doi:10.1371/journal.pone.0236622)

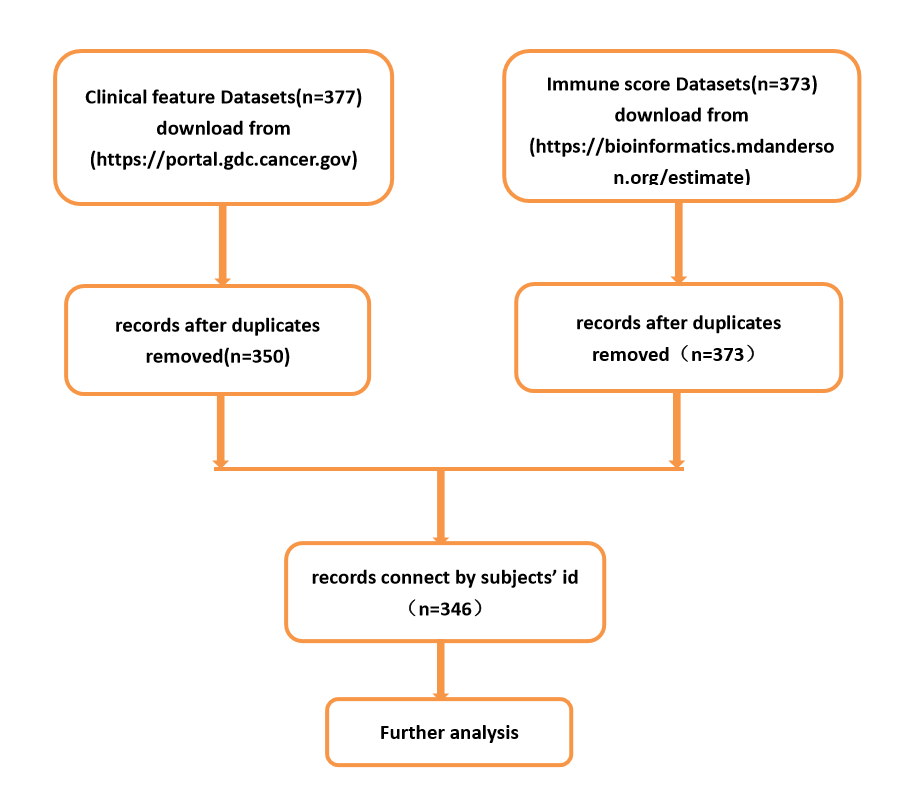

Supplement: S1 Fig — (TIF) [file pone.0236622.s001.tif]

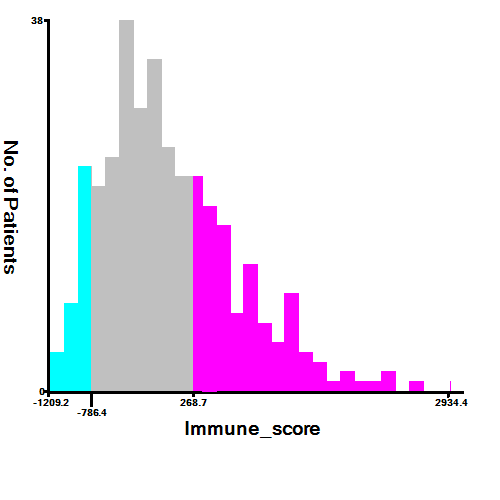

Supplement: S2 Fig — Low immune score subgroup:-1209.2 to -786.4; intermediate immune score subgroup:-786.4 to 268.7; high immune score subgroup:268.7 to 2934.4 (TIF) [file pone.0236622.s002.tif]

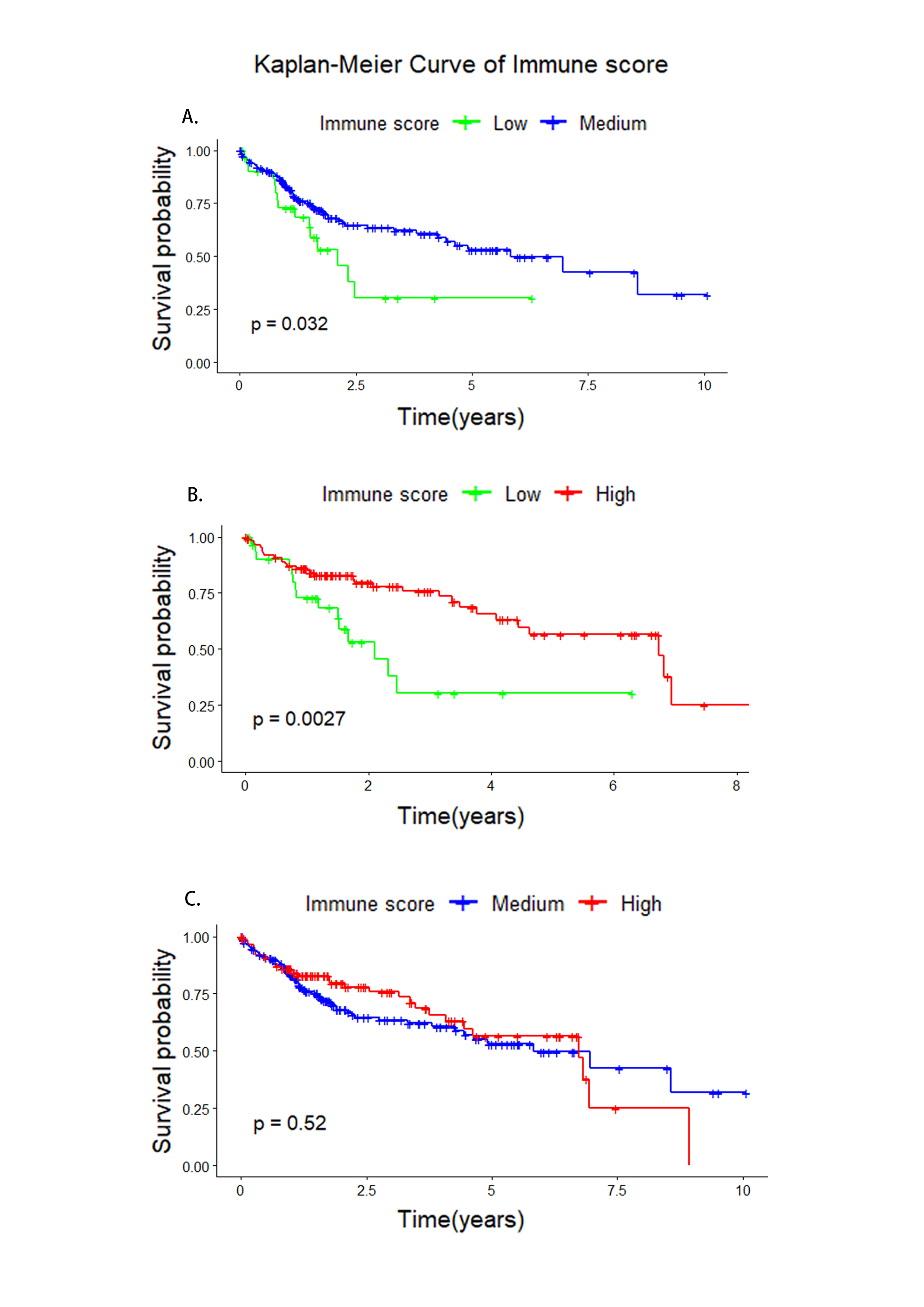

Supplement: S3 Fig — Kaplan–Meier curves depicting that in comparison with patients with low immune scores, those with intermediate and high immune scores had significantly improved survival time. p < 0.05; difference was statistically significant. (TIFF) [file pone.0236622.s003.tiff]

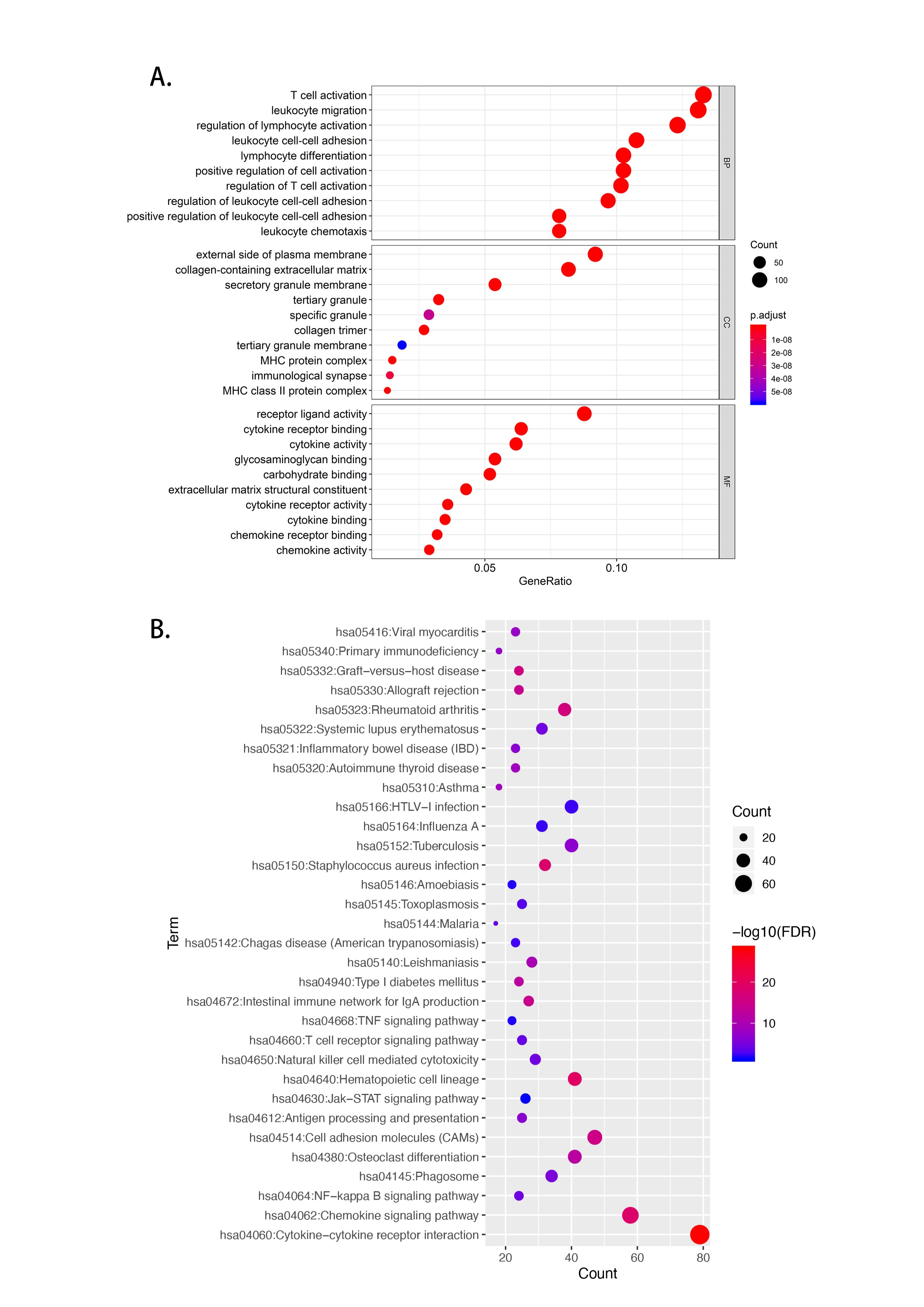

Supplement: S5 Fig — A. Gene ontology analysis: From top to bottom, the figure represents biological process, cellular component and molecular function, respectively. B. The most significant Kyoto Encyclopedia of Genes and Genomes (KEGG) pathways. The larger the circle, the more genes it contained; conversely, the smaller the circle, the fewer genes it contained. The color of the circle is correlated with the P-value. The smaller the P-value is, the closer it is to the red value. The larger the P-value is, the closer it is to the blue value. (TIFF) [file pone.0236622.s005.tiff]

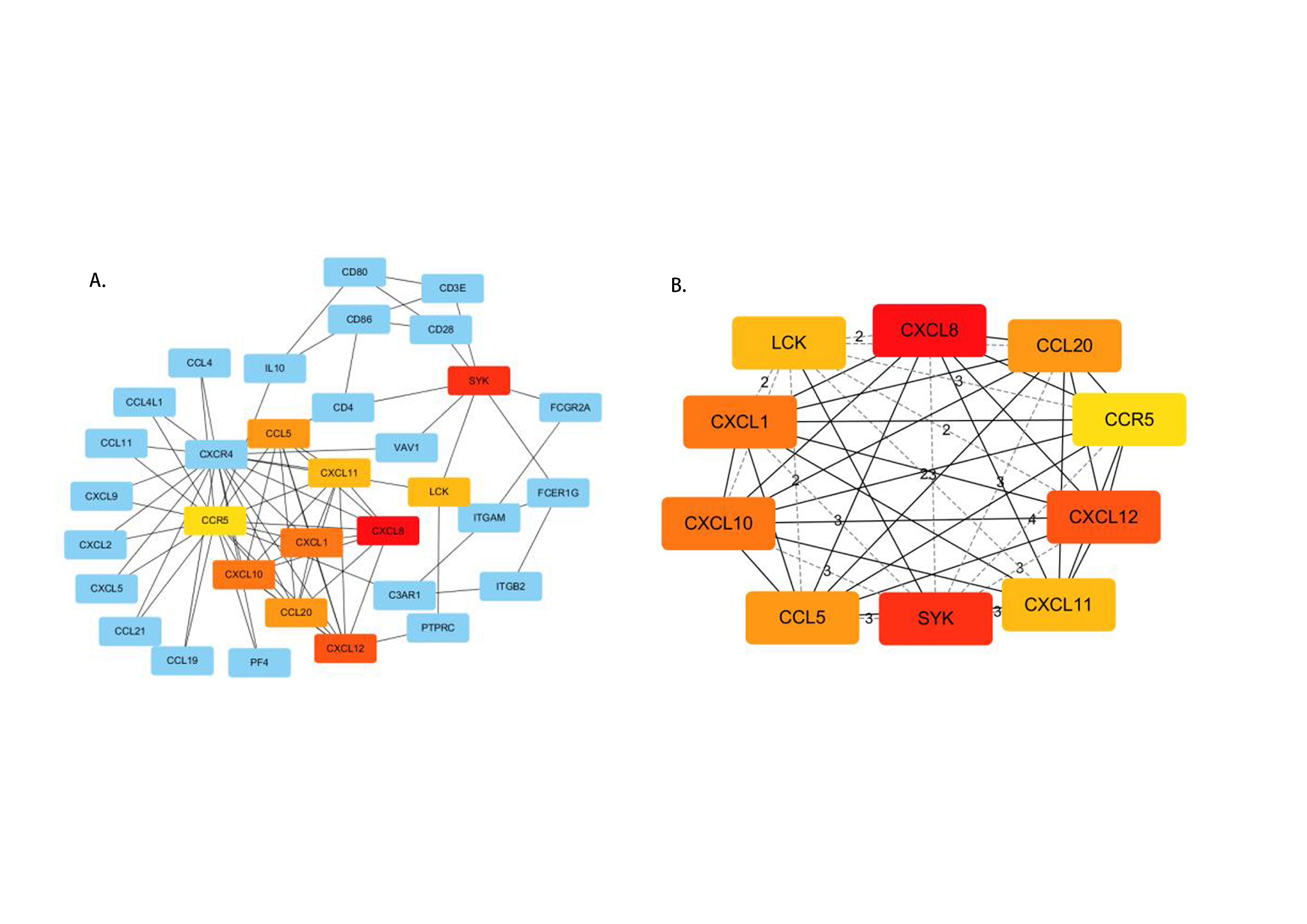

Supplement: S6 Fig — A. PPI network diagram of 33genes. B. The network diagram of top 10 hub genes. PPI–protein-protein interaction (TIFF) [file pone.0236622.s006.tiff]

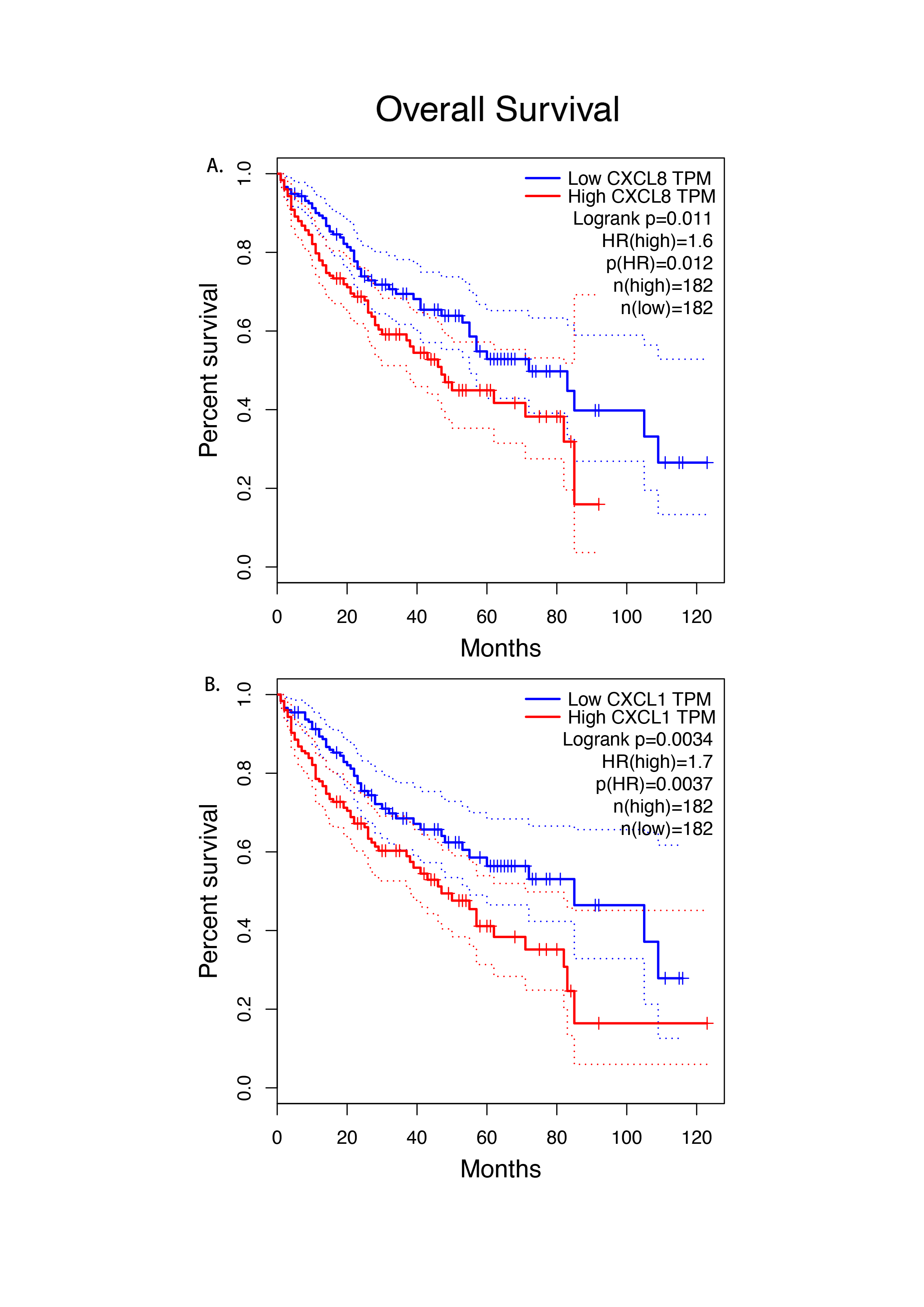

Supplement: S7 Fig — A. Overall survival of CXCL8. B. Overall survival of CXCL1. (TIFF) [file pone.0236622.s007.tiff]

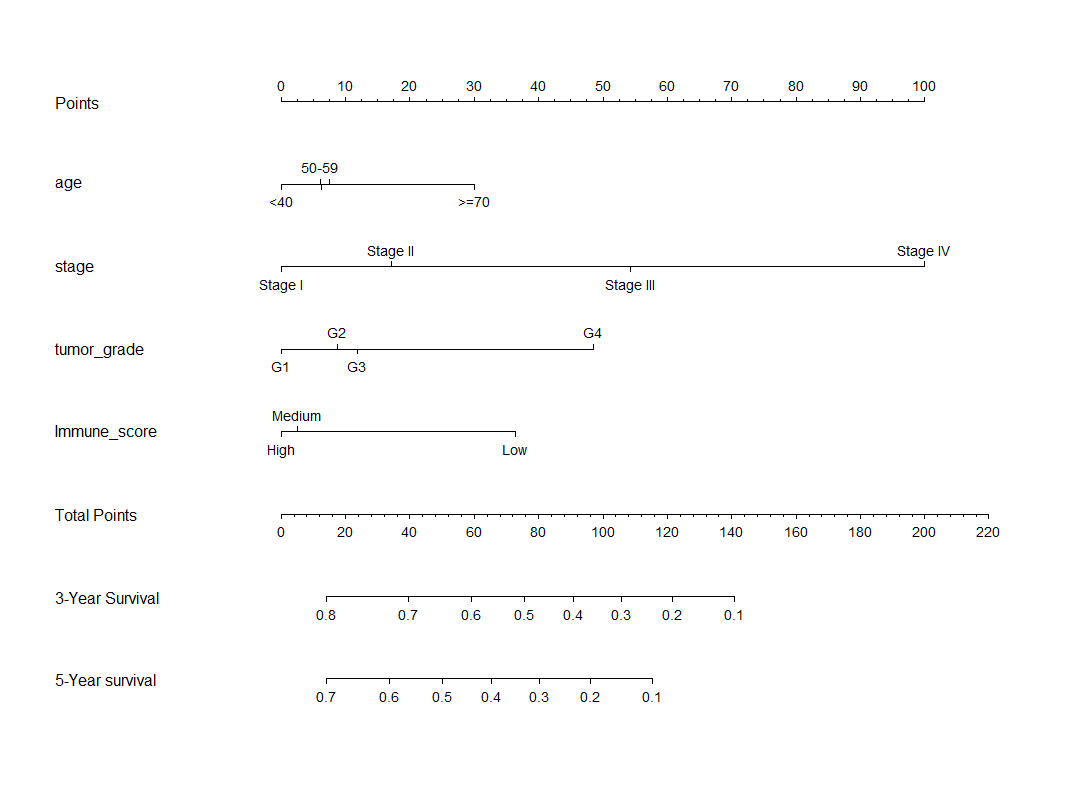

Supplement: S8 Fig — In these nomograms, each individual patient’s value is located on each variable axis and a line is drawn upward to determine the number of points received for each variable value. The sum of these numbers is placed on the total points axis and a line is drawn downward to the survival axes to determine the likelihood of three- or five-year survival. (TIFF) [file pone.0236622.s008.tiff]

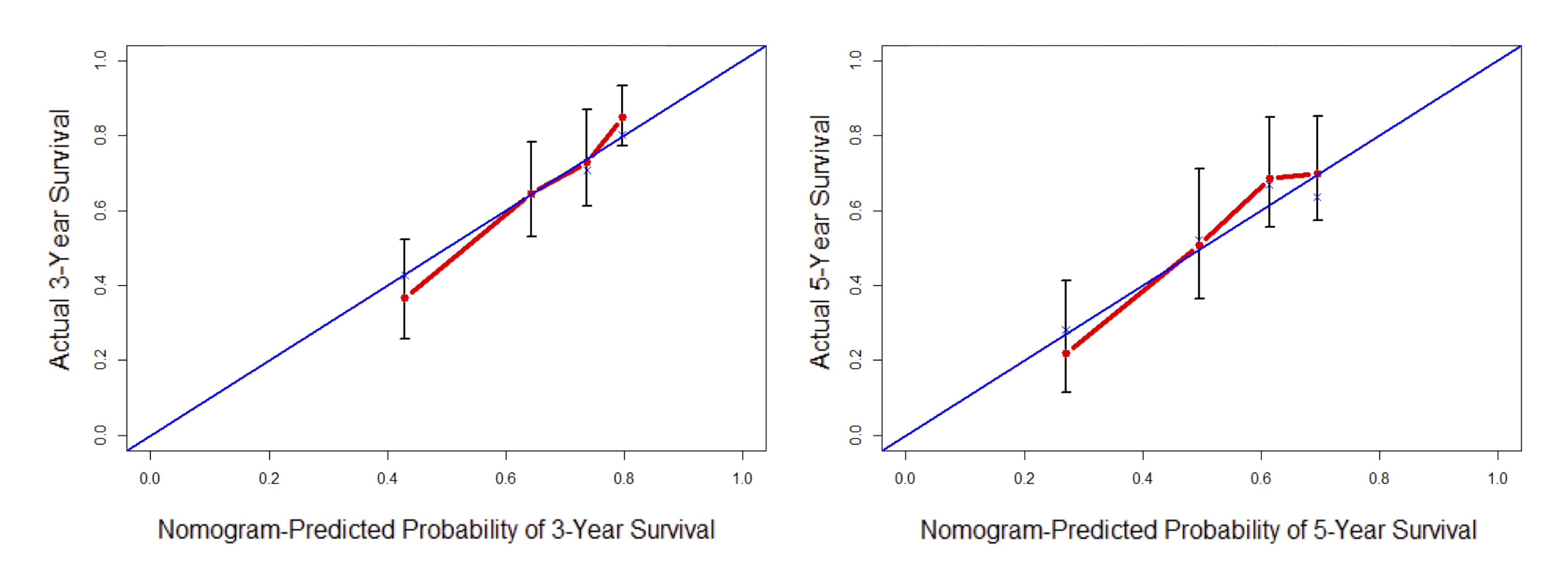

Supplement: S9 Fig — Nomogram-predicted probability of survival time is plotted on the x-axis; actual survival time is plotted on the y-axis. (TIFF) [file pone.0236622.s009.tiff]
