# Supplementary material for: A new clinical prognostic nomogram for liver cancer based on immune score
Source: PLoS One. 2020 Jul 30;15(7):e0236622. doi: 10.1371/journal.pone.0236622 (PMC7392298; doi:10.1371/journal.pone.0236622)
Supplement: Attachment — Submitted filename: Response to Reviewers.docx [file pone.0236622.s010.docx]

Dear Prof. Sai-Ching Jim Yeung

Thanks a lot for having reviewed our manuscript. Now we have revised the manuscript according to the academic editor and reviewers’ comments. Most of the revisions are in the manuscript.

**Response to the academic editor**

***Question1:*** Please ensure that your manuscript meets PLOS ONE's style requirements, including those for file naming. The PLOS ONE style templates can be found at https://journals.plos.org/plosone/s/file?id=wjVg/PLOSOne_formatting_sample_main_body.pdf and https://journals.plos.org/plosone/s/file?id=ba62/PLOSOne_formatting_sample_title_authors_affiliations.pdf

***Answer1:* We ensure that our manuscript meets PLOS ONE's style requirements.**

***Question2:*** Please provide the accession number of URL link to the specific TCGA dataset used in your study.

***Answer2:* We provide the accession number of URL link to the specific TCGA dataset used in our study.**

**Question3:** The nomogram should be applicable to the entire range of possible score. The maximum of total points is 215, not 200. The font size of the labeling on Figure 3 and Figure 6 should be larger

***Answer3:* We have made changes according to the above requirements of the editor.**

**Response to Reviewer #1:**

***Question 1:*** The proportional hazard assumption for multivariate Cox regression is questionable with crossing survival curves.

***Answer1:* Compared with the low immune score group, the p value of survival curve of medium or high immune scores group was less than 0.05, the difference was statistically significant. The cross of survival curve is mainly between the medium and high immune scores group, but there is no statistical significance between the medium and high immune scores groups. Kaplan–Meier curves depicting that in comparison with patients with low immune scores, those with medium and high immune scores had significantly improved survival time, so we think that the proportional hazard assumption for multivariate Cox regression is reliable.**

***Question 2:*** A comparison to other clinical nomograms without using immune score for liver cancer is needed.

***Answer2:*** **In clinic, we usually use tumor size, stage, lymph node metastasis to evaluate the prognosis of patients. Because of the lack of data about lymph node metastasis in TCGA data in this study, we did not build a clinical prediction model of tumor size, stage and lymph node metastasis for comparison. Our study aims to reveal the value of immune score in clinical prediction model and improve the basis for immunotherapy of liver cancer.**

***Question 3:*** It could be risky to apply the proposed nomogram based on the limited sample size in this study without external validation.

***Answer3:*** **Due to the small number of liver cancer samples in TCGA database, all data samples were in the experimental group and the validation group was not split. This is not only the deficiency of this study, but also the direction of the later study. Later, we plan to build a liver cancer data bank for further verification.**

**Response to Reviewer #2:**

***Question 1:*** Many statements throughout the manuscript lack appropriate references. In introduction section, authors should update most recent HCC clinical and basic research literatures. These should be added where appropriate. The ESTIMATE—a key method they used in this study to evaluate HCC samples but less described in details. Overall, the result section is also too simple and does not focus on what is the significance of the data on HCC study.

***Answer1:* In introduction section, we add some clinical immune related studies of liver cancer and elaborate the ESTIMATE. In the results section, by analyzing the expression of different genes in the groups with high and low immune scores, we can screen out the target genes that may guide the clinical prognosis of liver cancer to further enrich our results.**

***Question 2:*** TCGA also provide normal liver samples either adjacent liver tissues to liver tumor in same patient or nonmalignant liver tissues they collected. It is important to compare the immunoscore not only in HCC but also in nonmalignant liver tissues that may reflect whole liver tumor microenvironment and immunity in liver.

***Answer2:* As the reviewer said, if there is an immune score of adjacent liver tissues to liver tumor in same patient, it will better reflect the whole tumor microenvironment. Although TCGA database has case data of normal liver tissue, there is no case data of adjacent liver tissues to liver tumor in same patient, and TCGA only has corresponding clinical data of cancer cases; secondly, The ESTIMATE database only has corresponding immune score of cancer patients, so the current data can only reveal immune score of microenvironment of liver cancer.**

***Question 3:*** It is important to provide and describe what the novelty of the present study is, not only somewhat incremental given that this immunoscore has been shown in other many cancer types.

***Answer3:* We further analyzed and screened the immune genes related to the prognosis of liver cancer, and provided theoretical basis for the research of liver cancer clinical targeted drugs.**

***Question4:*** What are the specific immune or stromal makers or immune genes in this HCC data set, such as inflammation cytokines, TGF-beta genes, and exclusive/exhaustive T cell markers? They only show a general correlation between the Score and patient survival in HCC samples. It would strengthen to show some specific immune marker correlations. These data will provide important information for clinical use.

***Answer4:*** **Through the analysis of differential gene expression in the high and low immune score groups, we screened out 10 target genes, and further analyzed that a and B are related to the prognosis of liver cancer, so as to provide basis for the research and development of clinical targeted drugs for liver cancer.**

***Question5:*** Figure legend is too simple. The patients’ information they analyzed and the statistics should be included in. In the Fig.3, what is the detail method they used to get this p value 0.025? is it among 3 groups? Or only low group vs. high/medium groups? It looks like no significant between high and medium?

***Answer5:*** **Figure 3 has been modified.**
