# Supplementary material for: A new clinical prognostic nomogram for liver cancer based on immune score
Source: PLoS One. 2020 Jul 30;15(7):e0236622. doi: 10.1371/journal.pone.0236622 (PMC7392298; doi:10.1371/journal.pone.0236622)
Supplement: Attachment — Submitted filename: Response to Reviewers.docx [file pone.0236622.s011.docx]

Dear Prof. Sai-Ching Jim Yeung

Thanks a lot for having reviewed our manuscript. Now we have revised the manuscript according to the reviewers’ comments.

**Response to Reviewer #1:**

Answer1: Response to Reviewer#1 Question 1: Reviewer#1 is concerned about the proportional hazard assumption for the Cox regression model. Since Reviewer#1 raised the concern based on the appearance of the Kaplan-Meier curves, the authors should run diagnostic tests for Cox regression model: e.g.,

•Schoenfeld residuals to check the proportional hazards assumption

•Martingale residual to assess nonlinearity.

***Question 1: Thanks to the reviewer's reminder, the reviewer's intention was not fully understood at the beginning, which resulted in the failure to answer the reviewer's questions well. Now, according to the reviewer's suggestion, we use R language to test the proportional hazard assumption for the Cox regression model. The results show that for age, stage, tumor grade and immune score, P＞0.05.***

***Question 2:*** Response to Reviewer#1 Question 2: The inability to build a clinical prediction model for comparison should be discussed as a limitation of the study in the Discussion section.

***Answer2:*** ***We put this part of the deficiency in the discussion part of the article.***

***Question 3:*** Response to Reviewer#1 Question 3: Again, this limitation of small number liver cancer samples is another limitation of the study that should be mentioned in the Discussion section.

***Answer3:*** ***In the discussion part of the article, we will add some explanation on this point.***

**Response to Reviewer #2:**

***Question 1:*** Response to Reviewer#2 Question 2: Although the ESTIMATE database only has the corresponding immune scores of the cancer samples, the scores for normal tissue samples can be generated. The TCGA gene expression data for the normal liver samples can be downloaded. The ESTIMATE R packages can be downloaded at https://bioinformatics.mdanderson.org/public-software/estimate/. The ESTIMATE scores can be generated for the normal liver samples and compared.

***Answer1:*** ***The tumor microenvironment can be better reflected by the analysis of the immune score of the adjacent tissues of liver cancer. As the reviewer said, the immune score of the normal liver tissue sample data in TCGA can be obtained through the R package, but this part of the normal tissue sample is not the adjacent contrast tissue of the liver cancer tissue.***
